# Correction to “*GT1* regulates maize sex determination by affecting the jasmonate pathway”

**DOI:** 10.1111/tpj.71193

**Published:** 2026-10-29

Lin, Z. , Yang, J. , Liu, S. , Bai, Y. , Li, W. , Lai, J. , et al. (2025) 
*GT1* regulates maize sex determination by affecting the jasmonate pathway. The Plant Journal, 123, e70306. Available from: 10.1111/tpj.70306 40654294

In Figure S13, panel B, the second lane of the EMSA experiment was incorrectly labeled ‘GT1’. The correct label should be ‘**GT1** ^**V98M**^’ to accurately indicate the amino acid substitution of the mutant protein used in that binding reaction. The corrected Figure S13 is shown below.

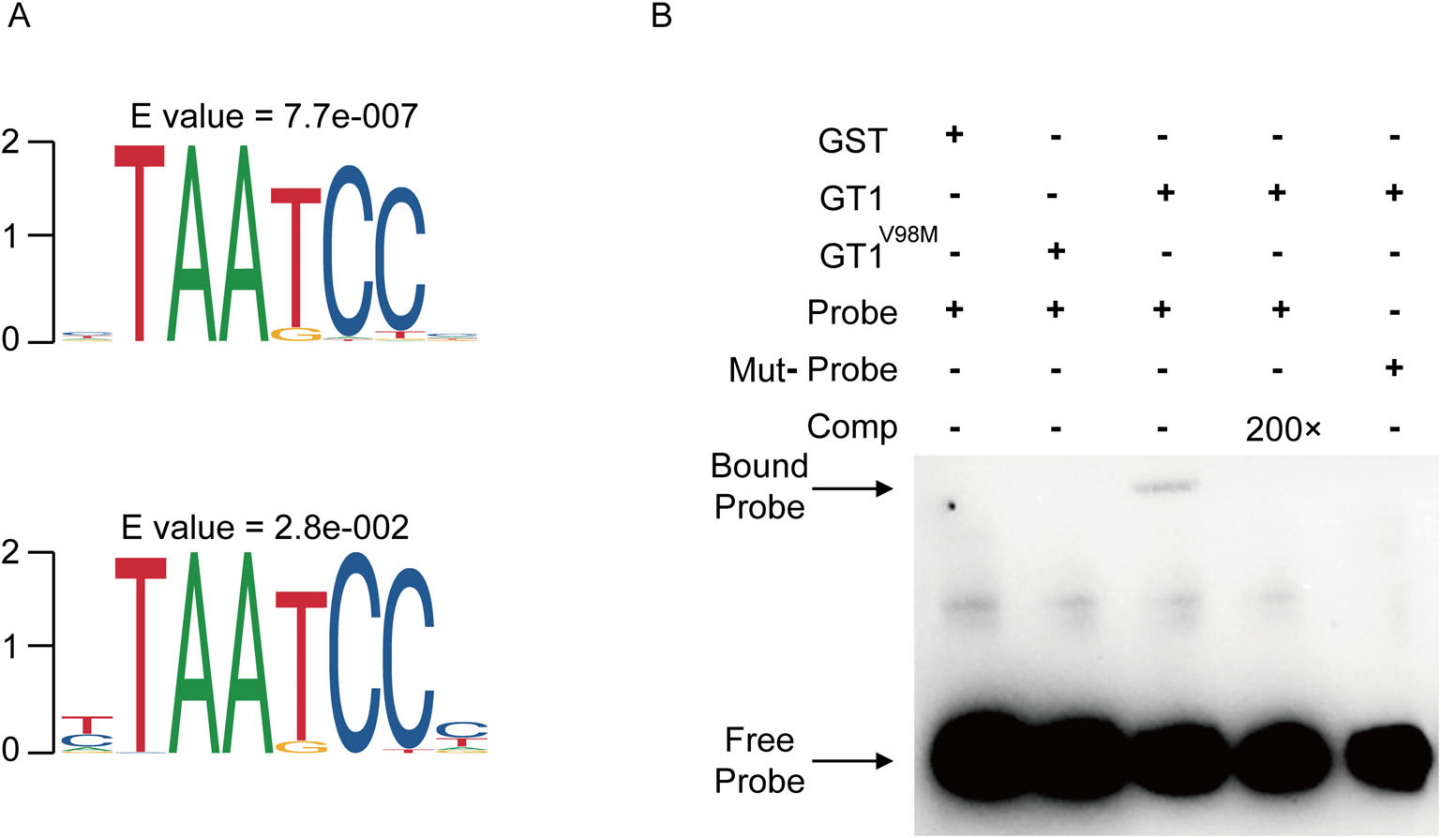

In the Results section, second paragraph, under the heading ‘*GT1* regulates maize sex determination by directly modulating JA biosynthesis’, the phrase ‘the primary active forms of JA’ should instead read ‘the primary forms of JA’. The word ‘active’ has been removed.

These corrections do not alter the original data, statistical analyses, or the conclusions of the article.

We apologize for these errors.

